# The occupational risk of *Helicobacter pylori *infection among gastroenterologists and their assistants

**DOI:** 10.1186/1471-2334-11-154

**Published:** 2011-05-31

**Authors:** Claudia Peters, Anja Schablon, Melanie Harling, Claudia Wohlert, José Torres Costa, Albert Nienhaus

**Affiliations:** 1 University Medical Center Hamburg-Eppendorf, Institute for Health Service Research in Dermatology and Nursing, Hamburg, Germany; 2 Occupational Health Division, Allergy and Clinical Immunology Division, Faculty of Medicine, Porto University, Porto, Portugal

## Abstract

**Background:**

*Helicobacter pylori *is a widely spread bacterium that mainly inhabits the gastric mucosa and can lead to serious illnesses such as peptic ulcer disease, gastric carcinoma and gastric MALT lymphoma. The oral-oral route seems to be the main transmission route. The fact that endoscopes are contaminated after being used to perform a gastroscopy leads one to question whether gastroenterologists and endoscopy nurses and assistants run a higher risk of infection.

**Methods:**

A systematic search for literature was conducted in the MEDLINE and EMBASE databases and further publications were found in reference lists of relevant articles. Epidemiological studies on the occupational exposure of endoscopy personnel were collected and their quality was assessed. Pooled effect estimates were identified in a meta-analysis.

**Results:**

Of the 24 studies included in the analysis, 15 were considered to be methodologically good. Of these 15 studies, eight single studies showed a statistically significant increased risk of infection for gastroenterologists, and five for their assistants. Meta-analysis across all methodologically good studies found a statistically significant risk of 1.6 (95%CI 1.3-2.0) for doctors. The pooled effect estimates also indicated a statistically significant risk of *Helicobacter pylori *infection (RR 1.4; 95%CI 1.1-1.8) for assistants too.

When studies are stratified by medical and non-medical control groups, statistically significant risks can only be recognised in the comparison with non-medical controls.

**Conclusions:**

In summary, our results demonstrated an increased risk of *Helicobacter pylori *infection among gastroenterological personnel. However, the choice of control group is important for making a valid assessment of occupational exposure risks.

## Background

*Helicobacter pylori *(*H. pylori*) is a gram-negative, spiral-shaped bacterium that mainly inhabits and multiplies in the gastric mucosa [1,2]. The bacterium produces the enzyme urease and this convert urea into carbon dioxide and ammonia. The ammonium envelope enables it to resist the acidic environment in the stomach [1]. The prevalence of *H. pylori *varies widely from region to region, with an especially marked difference between industrial and developing countries. The estimated prevalence in Asia is 50 to 80%, 30 to 50% in Western Europe and 30% in North America [3]. In Germany, the prevalence of *H. pylori *infections among the population is between 20 and 70% [4]. Within the framework of the German National Health Inverview and Examination Survey 1998 a 40% prevalence of *H. pylori *antibodies among the general population was found. In the youngest age group (aged 18 to 29) the prevalence was 22%, while in the oldest age group it was 61%. In the low socioeconomic status group, the prevalence was 51%, whereas it was 29% in the upper class [4]. There is a positive correlation between the number of persons in a household and the rate of *H. pylori *infection [5].

*H. pylori *is associated with peptic ulcer disease, distal gastric carcinoma and gastric MALT lymphoma [1]. It has long been known that the stomach harbours a population of bacteria [1], but not until Marshall and Warren's work in 1984 [6] was the link between Campylobacter pyloridis, as *H. pylori *was then known, and chronic gastritis appreciated, which "revolutionised the understanding of pathological gastric processes" [1]. Individuals infected with *H. pylori *run a three times greater risk of contracting an ulcus ventriculi and a two and a half times greater risk of developing adenocarcinoma of the stomach [7]. There also appears to be a synergistic carcinogenic effect when smoking and *H. pylori *infections are combined [8].

The transmission routes of *H. pylori *have yet to be fully explained [1,4,6]. The likelihood of infection increases with low social status and the associated crowded living conditions. The infection is mainly acquired in childhood [9]. *H. pylori *has been isolated from faeces [10], gastric juice, vomit, saliva and dental plaque [11,12]. It is transmitted from person to person. The oral-oral route seems to be the main route of transmission. Contact with regurgitated matter seems to play a more important role in transmission than contact with saliva, since promiscuity is not a transmission risk factor [2,7] and transmission between married couples is rare [13]. Dentists come into close contact with their patients' saliva, but although they have been found to be at greater risk [14], there is insufficient evidence of an increased risk of infection [15,16]. There are reports of a high prevalence of *H. pylori *infections in institutions for people with intellectual disability. Health care workers working in these institutions are especially vulnerable because of their close contact [17].

*H. pylori *inhabits the gastric mucosa. Consequently, the endoscopes used to perform gastroscopies on patients affected become contaminated. Infection can be passed on to other patients via these endoscopes. The first recorded nosocomial infection with *H. pylori *was reported in 1979. 17 of 37 healthy subjects who took part in a study on acid concentrations in the stomach developed gastritis after an endoscopy [18]. Although, appropriate decontamination can certainly prevent transmission via this route [18,19]. Given that the oral-oral route seems to be the predominant way of transmitting *H. pylori*, the question is whether doctors who perform gastroscopies, or the nurses who assist them, run an increased risk of infection. Several reviews have been published, but some data were controversial. We therefore checked the literature and conducted a meta-analysis to determine the occupational risk of *H. pylori *infection among gastroenterological personnel.

## Methods

### Search strategy and screening

For the literature search, we first considered the studies published in three review articles published in 1999 [20], 2001 [21] and 2004 [22]. This work was supplemented by a systematic search for literature in the MEDLINE and EMBASE databases using appropriate keywords - "Helicobacter pylori" combined with "occupational risk, endoscopy, gastroenterologist, healthcare worker" - for the years 1999 to 2010. We also searched through the reference lists of the chosen studies and included appropriate publications in our work.

The criteria for inclusion related to the following:

- Study design: cohort study or cross-sectional study

- Study population: the study investigates gastroenterologists and/or their assistants (nurses)

- Exposure: the study investigates occupation as a risk factor

- Languages: German and English

### Study quality

The methodological quality of the studies was assessed as moderate or good. A study was categorised as moderate if it did not take into account the potential confounding effect of age and socioeconomic status or did not adequately describe the control group's origin in order to check for the potential of confounding. Very small studies with fewer than 30 subjects per group also counted as methodologically moderate. Three authors carried out the literature screening and quality evaluation independently from one other and then compared their findings. Where they disagreed, a consensus was reached by means of discussion.

### Statistical analysis

The studies are differentiated into prospective incidence studies and retrospective prevalence studies. For the purposes of statistical analysis, we used the information on the number of gastroenterological personnel and the control group as a whole, and the proportion in each case that had tested positive for *H. pylori*. This data was used to calculate prevalence ratios as effect estimates in the case of retrospective original studies. These are described as relative risks (RR). 95% confidence intervals (95%CI) were generated to serve as statistical tests. For the purpose of meta-analysis, a combined effect estimate was calculated using the Mantel-Haenszel method for dichotomous outcomes. Stratification enabled us to conduct further differentiated analyses relating to individual occupational groups, the kind of controls, the study region and the time of publication.

### Heterogeneity

We carried out a chi-square test (χ^2^) in order to examine the statistical heterogeneity between studies. If there was statistically significant heterogeneity (P < 0.05) the random effect model was used to calculate the combined effect estimate, otherwise the fixed effect method was used.

### Sensitivity analysis

The impact of the studies on the combined effect estimate finding was tested by excluding individual studies from the analysis and examining the estimate stability [23]. The quality of the studies was also compared in this context. Where there is a clearly recognisable difference, the moderate studies can be considered separately or excluded from the analysis.

### Publication bias

We first showed a possible publication bias graphically using a funnel plot. The effect estimate was plotted versus the precision of the estimate (defined as the inverse of standard error (1/SE_i_)). An asymmetry of this funnel plot indicates publication bias. The funnel plot asymmetry was additionally measured following a linear regression approach on the natural logarithmic scale of the effect estimate. In this method, the standard normal deviate, defined as the effect estimate divided by its standard error, is regressed against the precision. The intercept provides a measure of asymmetry. The greater the deviation from zero, the stronger the evidence of asymmetry [24].

The analyses were carried out using Review Manager (RevMan 5) and Microsoft Excel.

## Results

### Studies identified and assessment of study quality

24 studies [25-48] dealing with occupational exposure to *H. pylori *among gastroenterologists and their colleagues were identified and included in our meta-analysis. The individual studies are listed in Tables 1 and 2. In the main, the study design is cross-sectional, so prevalence ratios are stated. The study by Hildebrand [27] is an exception. Here, the longitudinal design enables the authors to show incidence as well as prevalence. Thus, an incidence of 2.6% per year was found among gastroenterologists and an incidence of 0.14% per year among the control group. However, only retrospective prevalence analyses were taken into account in the meta-analysis.

**Table 1:** Information, risk estimation and quality assessment of studies with non-medical controls

First author, year	Study area	Gastros (HP positive %)	Controls (HP positive %)	Diagnostic method	RR	95%CI	Study quality
Mastromarino 2005 [25]	Italy	S 92 (40) D 47 (34) A 45 (37)	52 (19) hosp	Stool antigen test	S 1.9 D 1.8 A 2.1	1.04-3. 6 0.9-3.5 1.1-4.0	good

Birkenfeld 2004 [26]	Israel	S 190 (73) D 88 (72) A 50 (53)	4633 (53) pat	Breath test	S 1.8 D 1.4 A 1.5	1.3-1.5 1.2-1.5 1.3-1.7	good

Hildebrand 2000 [27]	Switzerland	D 92 (39)	168 (38) pop	Breath test	D 1.03	0.8-1.4	good

Ellett 1999 [28]	USA	A 138 (14)	112 (18) don	Serology	A 0.8	0.4-1.4	good

Monés 1999 [29]	Spain	D 137 (53)	189 (52) pop	Breath test	D 1.03	0.8-1.3	good

Abbas 1998 [30]	Pakistan	S 33 (79) D 19 (68) A 14 (93)	33 (58) neighbours	Serology	S 1.4 D 1.2 A 1.6	0.97-1.9 0.8-1.8 1.2-2.2	good

Nishikawa 1998 [31]	Japan	S 121 (30) D 92 (30) A 29 (28)	101 (25) pop	Serology	S 1.2 D 1.2 A 1.1	0.8-1.9 0.8-2.0 0.6-2.2	good

Braden 1997 [32]	Germany	S 1091 (38) D 922 (38) A 169 (37)	413 (27) pop	Breath test	S 1.4 D 1.4 A 1.4	1.2-1.7 1.2-1.7 1.1-1.7	good

Goh 1996 [33]	Malaysia	S 82 (33) D 34 (41) A 48 (27)	53 (11) pop	Breath test	S 2.9 D 3.6 A 2.4	1.3-6.6 1.6-8.5 0.99-5.8	good

Liu 1996 [34]	China	S 170 (81) D 125 (82) A 45 (78)	702 (45) pop	Serology	S 1.8 D 1.9 A 1.7	1.6-2.0 1.7-2.1 1.5-2.1	good

Chong 1994 [35]	USA	S 122 (53) D 111 (52) A 11 (52)	510 (14) don	Serology	S 3.8 D 3.7 A 3.9	2.9-5.0 2.8-4.9 2.2-6.9	good

Lin 1994 [36]	Australia	D 39 (69) A 107 (17)	195 (37) pop 115 (37) pop	Serology	D 1.9 A 0.6	1.4-2.5 0.4-1.01	good

Velasco 2007 [37]	Cuba	S 38 (39)	38 (8) hosp	Serology	S 5.0	1.6-15.9	moderate

Prónai 2000 [38]	Hungary	D 101 (30)	426 (54) pop	Breath test	D 0.6	0.4-0.8	moderate

Kamat 1999 [39]	India	D 17 (29)	35 (20) hosp	Serology	D 1.5	0.6-4.0	moderate

Rudi 1997 [40]	Germany	S 75 (24)	110 (35) hosp	Serology	S 0.7	0.4-1.1	moderate

Pristautz 1994 [41]	Austria	D 88 (57)	100 (51) pop+don	Serology	D 1.1	0.9-1.5	moderate

Mitchel 1989 [42]	Australia	S 101 (30) D 33 (51) A 68 (19)	715 (22) don	Serology	S 1.4 D 2.4 A 0.9	0.99-1.9 1.7-3.4 0.5-1.5	moderate

Reiff 1989 [43]	Germany	S 45 (69)	165 (65) stud+don+pat	Serology	S 1.1	0.9-1.3	moderate

Rawles 1987 [44]	USA	S 38 (32)	20 (10) don	Serology	S 3.2	0.8-12.8	moderate

Morris 1986 [45]	New Zealand	S 36 (25) D 21 (33) A 11 (18)	261 (37) pop	Serology	S 0.7 D 0.9 A 0.5	0.4-1.2 0.5-1.7 0.1-1.8	moderate

Gastros = gastroenterological staff

S = staff, D = doctors, A = assistants, don = blood donors, pat = patients, stud = students

hosp = hospital staff = without contact with patients or not defined

med = medical staff = in contact with patients

**Table 2:** Information, risk estimation and quality assessment of studies with medical controls

First author, year	Study area	Gastros (HP positive %)	Controls (HP positive %)	Diagnostic method	RR	95%CI	Study quality
Noone 2006 [46]	Scotland	A 74 (32)	148 (33)	Serology	A 0.98	0.7-1.5	good

Mastromarino 2005 [25]	Italy	S 92 (40) D 47 (34) A 45 (37)	105 (35)	Stool antigen test	S 1.05 D 0.97 A 1.1	0.7-1.5 0.6-1.6 0.7-1.8	good

Birkenfeld 2004 [26]	Israel	S 190 (73) D 88 (72) A 50 (53)	98 (70)	Breath test	S 1.02 D 1.00 A 1.02	0.9-1.2 0.9-1.2 0.8-1.3	good

Monés 1999 [29]	Spain	D 137 (53)	44 (50)	Breath test	D 1.07	0.8-1.3	good

Potts 1997 [47]	England/Wales	D 30 (50)	30 (10)	Breath test	D 5.0	1.6-15.5	good

Braden 1997 [32]	Germany	S 1091 (38) D 922 (38) A 169 (37)	604 (36)	Breath test	S 1.05 D 1.06 A 1.02	0.9-1.2 0.9-1.2 0.8-1.3	good

Su 1996 [48]	Taiwan	D 70 (80)	64 (52)	Serology	D 1.6	1.2-2.0	good

Goh 1996 [33]	Malaysia	S 82 (33) D 34 (41) A 48 (27)	25 (12)	Breath test	S 2.74 D 3.4 A 2.3	0.9-8.3 1.1-10.7 0.7-7.2	good

Prónai 2000 [38]	Hungary	D 101 (30)	108 (35)	Breath test	D 0.8	0.6-1.3	moderate

Kamat 1999 [39]	India	D 17 (29)	17 (18)	Serology	D 1.7	0.5-5.9	moderate

Rudi 1997 [40]	Germany	S 75 (24)	272 (35)	Serology	S 0.7	0.5-1.1	moderate

Mitchel 1989 [42]	Australia	S 101 (30) D 33 (51) A 68 (19)	35 (29)	Serology	S 1.04 D 1.8 A 0.7	0.6-1.9 0.97-3.4 0.3-1.4	moderate

Gastros = gastroenterological staff

S = staff, D = doctors, A = assistants

When study quality was assessed on the basis of the abovementioned criteria, 15 studies were categorised as good [25-36,46-48], and nine as methodologically moderate [37-45].

### Meta-analysis

Pooled analysis of all 24 studies included showed that gastroenterological staff (RR 1.34; 95%CI 1.14-1.58) exhibited a significantly increased risk of *H. pylori *infection. Pooling of all methodologically good studies confirmed this increased risk (RR 1.52; 95%CI 1.27-1.81), whereas the methodologically moderate studies provided no evidence of higher risk.

**Figure 1:**
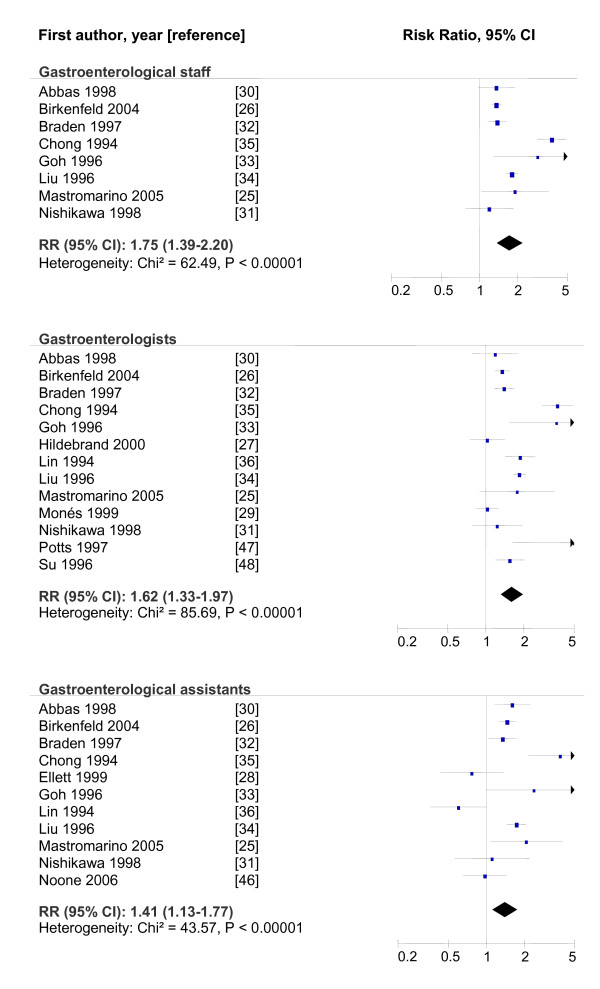
**Forest plots of methodologically good studies for gastroenterological personnel and the risk of *H. pylori *infection**. Block = risk estimates, line = 95% CI.

A differentiated analysis of the studies, which drew a difference between the information on gastroenterologists, their nurses/assistants and gastroenterological personnel in general, produced a similar picture. While the studies as a whole and the methodologically good studies (Figure 1) showed statistically significant results, the moderate studies showed no difference in *H. pylori *prevalence between gastroenterological personnel and the controls (Table 3).

**Table 3:** Summary of findings: Meta-analysis of H. pylori in gastroenterological personnel

	Number	Pooled estimation		Homogeneity	
	studies	RR	95% CI	**χ**^**²**^	p value
***All studies***					

All	24	1.34	1.14-1.58	174.51	< 0.00001
Staff	14	1.51	1.23-1.84	97.54	< 0.00001
Doctors	18	1.48	1.22-1.81	145.34	< 0.00001
Assistants	13	1.33	1.06-1.66	53.14	< 0.00001

***Good studies***					

All	15	1.52	1.27-1.81	100.00	< 0.00001
Staff	8	1.75	1.39-2.20	62.49	< 0.00001
Doctors	13	1.62	1.33-1.97	85.69	< 0.00001
Assistants	11	1.41	1.13-1.77	43.57	< 0.00001

***Moderate studies***					

All	9	1.04	0.77-1.41	34.13	< 0.00001
Staff	6	1.16	0.79-1.69	17.38	0.004
Doctors	5	1.12	0.62-2.03	41.25	< 0.00001
Assistants	2	0.80	0.50-1.28	0.72	0.4*

***Medical controls***					

Staff	6	1.02	0.90-1.15	6.30	0.28*
*good studies*	*4*	*1.06*	*0.96-1.18*	*3.17*	0.37*
Doctors	10	1.20	0.99-1.45	23.58	0.005
*good studies*	*7*	*1.21*	*0.98-1.50*	*19.00*	*0.004*
Assistants	6	1.04	0.91-1.20	3.66	0.6*
*good studies*	*5*	*1.07*	*0.93-1.23*	*2.06*	0.73*

***Non-medical controls***					

Staff	14	1.51	1.23-1.84	97.54	< 0.00001
*good studies*	*8*	*1.77*	*1.40-2.23*	63.90	*< 0.00001*
Doctors	16	1.41	1.13-1.77	159.35	< 0.00001
*good studies*	*11*	*1.55*	*1.22-1.96*	*100.13*	*< 0.00001*
Assistants	12	1.37	1.08-1.73	48.13	< 0.00001
*good studies*	*10*	*1.47*	*1.17-1.85*	*37.89*	*< 0.00001*

***Study area Europe***					

Staff	4	1.16	0.85-1.59	11.91	0.008
*good studies*	*2*	*1.43*	*1.21-1.69*	*0.97*	0.33*
Doctors	7	1.13	0.85-1.51	35.73	< 0.00001
*good studies*	*5*	*1.30*	*0.99-1.72*	*13.60*	*0.009*
Assistants	*3*				
*good studies*		*1.30*	*1.06-1.59*	*3.95*	0.14*

***Study area Asia***					

Staff					
*good studies*	*5*	*1.53*	*1.24-1.89*	*19.33*	*0.0007*
Doctors	7	1.53	1.26-1.86	18.97	0.004
*good studies*	*6*	*1.54*	*1.26-1.88*	*18.94*	*0.002*
Assistants					
*good studies*	*5*	*1.58*	*1.40-1.78*	3.97	0.41*

***Study area America / Australia***					

Staff (*1 good,5 moderate)*					
	6	2.07	0.98-4.40	41.01	< 0.00001
Doctors	4	2.08	1.30-3.34	21.65	< 0.00001
*good studies*	*2*	*2.63*	*1.34-5.20)*	*11.93*	*0.0006*
Assistants	5	0.99	0.47-2.10	29.24	< 0.00001
*good studies*	*3*	*1.21*	*0.36-4.03*	*28.02*	*< 0.00001*

***Diagnostic method: breath test***					

Staff	3	1.41	1.22-1.63	3.46	< 0.00001
Doctors	7	1.23	0.92-1.64	46.31	< 0.00001
Assistants	3	1.45	1.24-1.69	1.55	0.46*

***Diagnostic method: serology***					

Staff	10	1.47	1.07-2.04	84.87	< 0.00001
Doctors	11	1.66	1.33-2.09	53.62	< 0.00001
Assistants	9	1.17	0.79-1.73	57.54	< 0.00001

***Publication date ≤ 1989***					

Staff	4	1.14	0.94-1.38	6.67	0.08*
Doctors	2	1.30	0.33-5.05	15.56	< 0.00001
Assistants	2	0.80	0.50-1.28	0.72	0.4*

***Publication date 1990 - 1999***					

Staff	7	1.62	1.17-2.24	61.13	< 0.00001
Doctors	12	1.66	1.32-2.08	80.05	< 0.00001
Assistants	8	1.42	1.02-1.98	38.77	< 0.00001

***Publication date ≥ 2000***					

Staff	3	1.93	1.05-3.54	6.87	0.03
Doctors	4	1.04	0.62-1.76	36.22	< 0.00001
Assistants	3	1.36	1.15-1.61	5.19	0.07*

* Fixed effect model

The studies chose to use different control groups, recruiting them either directly from the population at large or non-medical fields ( = without contact with patients, Table 1), or from hospital staff from other departments ( = in contact with patients, Table 2). When the studies are stratified by non-medical controls, pooled analysis showed statistically significant risks for all personnel (RR 1.74; 95%CI 1.23-2.48), for doctors (RR 1.39; 95%CI 1.09-1.77) and for nurses/assistants (RR 1.37; 95%CI 1.08-1.74). In contrast, comparison with medical controls revealed no statistically significant risks.

When the studies were differentiated by location in Europe, Asia and America/Australia, studies conducted in Asia showed a marked 50% increase in risk for all occupational groups in gastroenterological departments. Assistants were the only group observed in the European studies to be at significantly higher risk statistically, whereas doctors were at significantly higher risk in America/Australia. If only methodologically good studies are considered, there is no change in the Asian studies, whereas in Europe a statistically significant increase in risk for all personnel can be seen.

Stratification by method of diagnosis revealed no striking differences between breath test diagnosis and diagnosis by serological examination. Stratification by date of publication showed significantly higher risks for the period 1990-1999 in all groups statistically, while no increase in risk could be seen prior to 1990. For studies published in 2000 or later, statistically significant risks could be seen for endoscopy personnel in general, and for nurses/assistants.

### Heterogeneity and sensitivity analysis

Testing for homogeneity revealed clear evidence of heterogeneity among most studies included in this paper. Accordingly, the random effect model was used to calculate the pooled effect estimate. A sensitivity analysis was also carried out, excluding individual studies from the meta-analysis one by one so as to show their influence on the pooled effect estimate. The relative risks thus calculated ranged from 1.26 to 1.40 and were all statistically significant.

### Publication Bias

The funnel plot showed no evidence of a marked publication bias (funnel plot not shown), nor did linear regression reveal any significant funnel plot asymmetry (intercept 0.59; 95%CI -1.95-1.83).

## Discussion

Pooled analysis of 24 retrospective prevalence studies revealed a higher risk of *H. pylori *infections among endoscopy personnel, which was statistically significant. This risk is evident for gastroenterologists and for their assistants. The composition of the control group was also found to have a considerable impact. The inclusion of controls that were from the general population or were at least non-medical highlighted significant differences in *H. pylori *prevalence. On the other hand, comparison with medical controls whose occupational exposure was characterised by contact with patients failed to confirm an increased risk for gastroenterological personnel.

Methodologically good studies were more likely to show statistically significant risks than studies of moderate quality. The use of inadequate controls or insufficient adjustment for confounders like age and socioeconomic status seemingly diluted the effect estimates in studies with moderate quality.

### Study area

With respect to the study area, the question arises why the results showed clear levels of significance only in Asia, and not in Europe or America/Australia. The number of studies and their populations cannot account for this, given that more gastroenterological personnel were examined in Europe than in Asia (Table 4). Nor can the quality of studies be sufficient reason for this difference, although stratification by study quality did reveal larger effect estimates in methodologically good studies than in those with moderate quality in Europe. *H. pylori *prevalence in the present studies differs very widely as reflected in the individual study regions. The median prevalence in Europe is 39% among doctors performing gastroscopies and 37% for their assistants. In contrast and somewhat surprisingly, the prevalence among non-medical control groups (studies N = 8) is 45%, in contrast with the 35% prevalence among medical controls (studies N = 7). The prevalence among doctors and nurses in Asia is significantly higher by comparison (median 68% and 78% respectively), whereas among the control groups it is considerably lower (both 35%). There is also a clear difference between target population and controls in the studies from America/Australia, albeit at a much lower level - doctors 52%, assistants 18%, non-medical controls 16%, medical controls 29%. The different *H. pylori *prevalence rates of the general population in different regions (Asia 50-80%, Europe 30-50%, North America 30% [3]) corroborate the findings in the non-medical controls. However, why the present studies from Europe found a higher prevalence among non-medical controls than among gastroenterological personnel remains obscure.

**Table 4:** Regional distribution of studies

Study area	Number studies	Quality	Gastro staff (HP positive %)	Doctors (HP positive %)	Assistants (HP positive %)	Non-medical controls (HP positive %)	Medical controls (HP positive %)
**Europe**							

	10	all	1303 (37)	1417 (39)	288 (37)	1623 (45)	1311 (35)
	6	good	1183 (37)	1228 (39)	288 (37)	822 (33)	931 (35)
	4	moderate	120 (46)	189 (43)		801 (53)	380 (35)
**America / Australia**							

	7	all	481 (31)	204 (52)	335 (18)	1929 (16)	35 (29)
	3	good	268 (42)	150 (61)	256 (17)	895 (16)	
	4	moderate	213 (31)	54 (42)	79 (19)	1034 (16)	35 (29)
**Asia**							

	7	all	596 (73)	445 (68)	186 (78)	5557 (35)	204 (35)
	6	good	596 (73)	428 (70)	186 (78)	5522 (45)	187 (52)
	1	moderate		17 (29)		35 (20)	17 (18)

Gastro staff = gastroenterological staff

### Diagnostic method

In epidemiological studies, a *H. pylori *infection is mainly diagnosed by non-invasive methods such as serology or breath tests. More rarely, a stool antigen test is used. In the present studies this method was used to ascertain prevalence in only one study. In contrast, 16 studies used serological tests and seven used breath tests. After stratification by diagnostic method, the meta-analysis showed no differences in risk. The main advantage of serology is that it is a quick, low-cost method for use with large study populations. Nonetheless, this method has various limitations. These relate in part to defining a cut-off value to differentiate between positive and negative results and to the sensitivity of the test to changes in reagents and laboratory conditions, which is particularly relevant in longitudinal studies and repeat examinations. The breath test (C13-UBT) is somewhat more cost-intensive, but its sensitivity and specificity are in the order of 90-95%, and this method is just as suitable as the stool test, particularly when examining children [49,50].

### Comparison with other studies

Since the *H. pylori *bacterium was discovered, numerous reviews have been published on the subject. Some dealt with the occupational risk of infection run by medical personnel in general, while some examined gastroenterological personnel in particular. Matysiak-Budnik [51] showed an association between occupational exposure and an increased risk of infection. Williams [20], too, stated that there were increased occupational risks for endoscopy personnel. However, the evidence in this review appears contradictory, since the findings varied between no risk and a five times greater risk. De Schryver et al. [21,22] were able to show in their reviews increased risks for gastroenterologists and endoscopy personnel. Magalhaes Queiroz [52] found controversial data on the occupational risk, but they considered only some of the studies also included here for gastroenterological personnel.

### Strength and limitations of the review

To our knowledge, this is the only meta-analysis to date to focus on the prevalence of *H. pylori *infection among endoscopy personnel. The strengths of this work are those of a meta-analysis. A meta-analysis can give a comprehensive overview of the state of research. Pooling different studies on a topic increases statistical power and validity in comparison with smaller individual studies and increases the likelihood of being able to identify actual differences that exist between groups [53]. Nonetheless, this form of study also has limitations, and the result of the effect estimate can only be considered and interpreted in relation to the underlying data. One problem in this paper is the statistical heterogeneity among studies. This was taken into account first by applying the random effect model. In addition, sub-group analyses were carried out so that only studies with identical characteristics were pooled and analysed [23].

The search strategy to listed papers might introduce publication bias, because inconclusive studies might be less likely published in listed journals. However, we controlled for publication bias and found no evidence for it.

An adequate control for confounding is crucial in occupational infectious disease epidemiology, especially when transmission of infection mainly occurs in childhood. Therefore, for a study to be classified as good, it needed to give the confounding nature of age and social status due consideration. However, the control for significant confounders via selection of the control group or by adjustment could not be assessed for effectiveness.

Although the assessment of study quality was taken into account, further criteria must be heeded when pooling and calculating pooled estimates - differences in risk factors, in the observance or adjustment of confounders, and in the study population (different regions, different survey dates, different study approaches/control groups). A lack of information about age, gender, ethnicity and work-related factors, such as the use of gloves and masks or the frequency of gastroscopies performed, precludes more differentiated analyses.

## Conclusions

Gastroenterological personnel are exposed to an increased risk of *H. pylori *infection. Further studies involving suitable control groups and designed as prospectively as possible should identify the precise risk.

## Competing interests

The authors declare that they have no competing interests.

## Authors' contributions

AS made substantial contributions to the design of the study and to the extraction and interpretation of data. She was involved in the drafting of the major scientific content in the manuscript. MH made substantial contributions to the analysis and interpretation of the data. She was involved in proofreading and editing the scientific content of the manuscript. CW made substantial contributions to the design of the study. She was involved in proofreading and editing the scientific content of the manuscript. JTC made substantial contributions to the interpretation of data and was involved in revising the manuscript critically for important intellectual content. AN made substantial contributions to the design and to the extraction and interpretation of data. He was involved in drafting the manuscript. CP made substantial contributions to the design of the study, the extraction of data, and to the analysis and interpretation of data. She was involved in drafting the manuscript. All authors confirm that they have seen and approved the final version and have no conflicts of interest.

## Pre-publication history

The pre-publication history for this paper can be accessed here:

http://www.biomedcentral.com/1471-2334/11/154/prepub
